# Azithromycin-resistant *Neisseria gonorrhoeae* isolates in Guangzhou, China (2009–2013): coevolution with decreased susceptibilities to ceftriaxone and genetic characteristics

**DOI:** 10.1186/s12879-016-1469-3

**Published:** 2016-04-14

**Authors:** Jing-Yao Liang, Wen-Ling Cao, Xiao-Dong Li, Chao Bi, Ri-Dong Yang, Yan-Hua Liang, Ping Li, Xing-Dong Ye, Xiao-Xiao Chen, Xi-Bao Zhang

**Affiliations:** Institute of Dermatology, Guangzhou Medical University, Guangzhou, 510095 PR China; Department of Dermatology, Guangzhou Institute of Dermatology, 56 Hengfu Road, Guangzhou, 510095 PR China

**Keywords:** *Neisseria gonorrhoeae*, Azithromycin, Antimicrobial resistance, NG-MAST

## Abstract

**Background:**

The recent emergence of azithromycin-resistant (AZM-R) *N. gonorrhoeae* isolates that have coevolved decreased susceptibility to extended-spectrum cephalosporins has caused great concern. Here we investigated the prevalence of decreased susceptibility to ceftriaxone (CRO^D^) in AZM-R isolates and genetically characterized AZM-R isolates in Guangzhou, China from 2009 to 2013.

**Methods:**

The minimum inhibitory concentration (MIC) of AZM and ceftriaxone was determined using an agar-dilution method. All AZM-R isolates were screened for mutations in 23S rRNA, *mtrR* and penA genes and genotyped using *N. gonorrhoeae* multi-antigen sequence typing (NG-MAST).

**Results:**

Of the 485 identified *N. gonorrhoeae* isolates, 445 (91.8 %) were isolated from male urethritis subjects, and 77 (15.9 %) were AZM-R (MIC ≥ 1 mg/L), including 33 (6.8 %) with AZM low-level resistant (AZM-LLR, MIC = 1 mg/L) and 44 (9.1 %) with AZM middle-level resistant (AZM-MLR, MIC ≥ 2 mg/L). Significantly more CRO^D^ (MIC ≥ 0.125 mg/L) showed in AZM-MLR isolates (43.2 %, 19/44) as compared with that in AZM-LLR isolates (18.2 %, 6/33) (*p* < 0.05). For the 23S rRNA, *mtrR*, penA or combined 23S rRNA/*MtrR*/penA mutations, no significant difference was found between AZM-LLR isolates and AZM-MLR isolates (*P* > 0.05); similar results were detected between combined AZM-LLR/CRO^D^ isolates and combined AZM-MLR/CRO^D^ isolates (*P* > 0.05). No mutation A2059G or AZM high-level resistant (AZM-HLR, MIC ≥ 256 mg/L) isolate was detected. Among 77 AZM-R isolates, 67 sequence types (STs) were identified by NG-MAST, of which 30 were novel. Most STs were represented by a single isolate.

**Conclusions:**

The AZM-R together CRO^D^ isolates are now present in Guangzhou, China, which deserve continuous surveillance and the mechanism of concurrent resistance needs further study.

**Electronic supplementary material:**

The online version of this article (doi:10.1186/s12879-016-1469-3) contains supplementary material, which is available to authorized users.

## Background

The global spread of multidrug-resistant *Neisseria gonorrhoeae* (*N. gonorrhoeae*) is a growing public health threat. Worryingly, clinical treatment failures with the extended-spectrum cephalosporins (ESCs) [1], the last remaining options for empirical first-line monotherapy, have recently been reported. Accordingly, dual antimicrobial therapies have been introduced in several countries [2, 3], including China [4]. These consist of ceftriaxone (250–500 mg intramuscularly) or cefixime (400 mg orally, if ceftriaxone is not an option) together with azithromycin (AZM, 1–2 g orally) for treatment of uncomplicated gonorrhea. AZM was recommended for patients with gonorrhea or coinfection with *Chlamydia trachomatis* in China around the year 2000 [5], and since then has been broadly used because of its wide availability and ease of administration. In China, AZM-resistant (AZM-R) *N. gonorrhoeae* were first identified during 2001–2003 [6], and the first AZM-R isolates were identified in Guangzhou in 2009 [7]. Treatment failures with 2 g of AZM and high-level AZM resistant *N. gonorrhoeae* isolates with a minimum inhibitory concentration (MIC) ≥ 256 mg/L have been verified in several countries [8, 9]. Recently, a surveillance of antimicrobial resistance in gonococci from Canada resulted in two high-level AZM-R isolates (MIC ≥ 2048 mg/L), which may be due to mutation A2143G (*N. gonorrhoeae* numbering) in the four copies of the 23S rRNA gene [9].

Mutations at various genes such as 23S rRNA, *mtrR* and penA (encoding penicillin-binding protein 2, PBP2), have been identified associating with chromosomally mediated resistance to AZM [10, 11] and ESCs [12] in *N. gonorrhoeae*. Globally, AZM-R together decreased susceptibility to ESCs in *N. gonorrhoeae* has only rarely been reported [9, 13]. In this study, we investigated the prevalence of decreased susceptibility to ceftriaxone (CRO^D^) in AZM-R *N. gonorrhoeae* isolates, and the molecular characteristics of AZM-R *N. gonorrhoeae* isolates from January 2009 to November 2013 in Guangzhou, China.

## Methods

### Gonococcal isolates and susceptibility testing

Clinical *N. gonorrhoeae* isolates were consecutively collected from urethral or cervical specimen of one per patient with gonorrhea attending sexually transmitted infection (STI) clinics in Guangzhou, China between January 2009 and November 2013. The demographic and clinical information of patients such as gender, age, and symptoms was collected by completing a questionnaire. Each isolate was cultured, verified, and preserved as described [14]. MIC for AZM and ceftriaxone were determined by the agar dilution method, according to recommendations from the WHO [15]. Antimicrobial susceptibility for AZM was interpreted using the European Committee on Antimicrobial Susceptibility Testing (EUCAST) guidelines (http://www.eucast.org/), and ceftriaxone susceptibility was interpreted according to criteria defined by WHO in 2012 [15]. Briefly, isolates with MIC ≥ 1.0 mg/L for AZM were classified as resistant, and isolates with MIC ≥ 0.125 mg/L for ceftriaxone were classified as having decreased susceptibility. *N. gonorrhoeae* ATCC 49226 and strains G, L, and P of the 2008 WHO *N. gonorrhoeae* reference strain panel were used as quality control strains. To compare with a previous report [5] and better analyze the results, AZM-R *N. gonorrhoeae* isolates (MIC ≥ 1.0 mg/L) were divided into AZM low-level resistant (AZM-LLR) *N. gonorrhoeae* isolates (MIC = 1.0 mg/L), AZM middle-level resistant (AZM-MLR) *N. gonorrhoeae* isolates (MIC ≥ 2.0 mg/L), and AZM high-level resistant (AZM-HLR) *N. gonorrhoeae* isolates (MIC ≥ 256 mg/L) in this study based on the previous studies [9, 10].

### Sequence-based molecular epidemiologic studies

Bacterial genome DNA from each identified AZM-R isolate was extracted using the TIANamp Bacterial DNA kit (TIANGEN, Beijing, China). As previously described, mutations in the four alleles of the 23S rRNA [16], *mtrR* and penA genes [12], and polymorphisms in *porB* and *tbpB* [17] were amplified by PCR. The PCR products were purified and sequenced by Shenggong Co., Ltd. (Shanghai, China). DNA sequences were aligned using BLAST and GenBank programs (http://www.ncbi.nlm.nih.gov) to identify mutations in the 23S rRNA gene. DNA sequences of *mtrR* and penA genes were translated into deduced amino acid sequences, which were then aligned with their respective prototypes in *N. gonorrhoeae* using Proteomics and Sequence Tools (http://ca.expasy.org/). *N. gonorrhoeae* isolates were typed using the *N. gonorrhoeae* multi-antigen sequence typing (NG-MAST) method [17]. NG-MAST allele numbers of *porB* and *tbpB* and STs were obtained using a publicly accessible database on the NG-MAST website (www.ng-mast.net). A phylogenetic tree was created using ClustalX software (version 1.83; http://www.clustal.org/download/1.X/ftp-igbmc.u-strasbg.fr/pub/ClustalX. html) and Mega software (version 6.0.5; http://www.megasoftware.net/mega.php) based on the more variable segments of *porB* (490 bp) and *tbpB* (390 bp) as analyzed by NG-MAST.

### Statistical analysis

Statistical significance was assessed using SPSS 13.0 (SPSS Inc, Chicago, IL). Chi-square tests were used for statistical analyses. A *P*-value < 0.05 was considered to be significant.

### Ethics approval

The study protocol was approved by the Medical Ethics Committee at the Institute of Dermatology, the Chinese Academy of Medical Sciences & Peking Union Medical College and the National Center for Sexually Transmitted Disease Control, Nanjing, China (approval number 2011-KY-003). Written informed consent was obtained from all participating subjects.

## Results

### Antimicrobial susceptibility testing

**Fig. 1:**
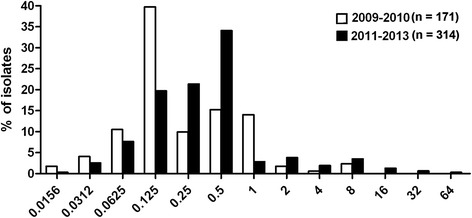
Distribution of MIC of AZM-R *N. gonorrhoeae* isolates during 2009 and 2010 (*n* = 171) versus 2011–2013 (*n* = 314)

A total of 485 *N. gonorrhoeae* isolates were identified in Guangzhou from 2009 to 2013 (Table 1). Of these, the mean age of the patients was 31.6 years (range from 17 to 69 years), 91.8 % (445/485) isolates were isolated from male patients with urethritis. Among all the isolates, 15.9 % (77/485) showed AZM-R, including 6.8 % (33/485) AZM-LLR and 9.1 % (44/485) AZM-MLR. The proportion of AZM-LLR isolates decreased from 14.0 % (24/171) in 2009–2010 to 2.9 % (9/314) in 2011–2013 (*p* < 0.05), whereas AZM-MLR isolates increased from 4.7 % (8/171) in 2009–2010 to 11.5 % (36/314) in 2011–2013 (*p* < 0.05). In the 2011–2013 study period, seven isolates with MIC > 8 mg/L appeared for the first time and accounted for 2.2 % (7/314) of the examined isolates (Fig. 1). The percentage of CRO^D^ isolates was 22.3 % (108/485) in all identified *N. gonorrhoeae* isolates, 18.2 % (6/33) in AZM-LLR isolates, and 43.2 % (19/44) in AZM-MLR isolates which was a significant increase compared with the percentage of all identified *N. gonorrhoeae* isolates or AZM-LLR isolates (both *p* < 0.05, Table 2).

**Table 1:** The resistance rates for AZM in *N. gonorrhoeae* isolated in Guangzhou, China between 2009 and 2013

	MIC		
Year	1 mg/L	≥ 2 mg/L	Total
2009	19.3 % (17/88)	4.5 % (4/88)	23.9 % (21/88)
2010	8.4 % (7/83)	4.8 % (4/83)	13.3 % (11/83)
2011	0.9 % (1/114)	7.9 % (9/114)	8.8 % (10/114)
2012	2.0 % (2/100)	17.0 % (17/100)	19.0 % (19/100)
2013	6.0 % (6/100)	10.0 % (10/100)	16.0 % (16/100)
Total	6.8 % (33/485)	9.1 % (44/485)	15.9 % (77/485)

**Table 2:** Characteristics of *N. gonorrhoeae* isolates with AZM-R and CROD identified in Guangzhou in 2009-2013

	AZM-LLR (*n* = 33)		AZM-MLR (*n* = 44)		
	Total^c^	CRO^D^	Total^c^	CRO^D^	
	*n* (%)	*n* (%)	*n* (%)	*n* (%)	*P*
Antimicrobial resistance	33 (100.0)	6 (18.2)	44 (100)	19 (43.2)	b < 0.05
23 S rRNA					
WT	32 (97.0)	5 (15.2)	38 (86.3)	13 (29.5)	a, b > 0.05
C2611T^S^	1 (3.0)	1 (3.0)	1 (2.3)	1 (2.3)	
C2611T^All^	0 (0.0)	0 (0.0)	5 (11.4)	5 (11.4)	
*mtrR*					
Deletion^a^					
Yes	29 (87.9)	6 (18.2)	42 (95.5)	19 (43.3)	a > 0.05, b: ND
No	4 (12.1)	0 (0.0)	2 (4.5)	0 (0.0)	
Mutations					
WT^b^	2 (6.1)	0 (0.0)	2 (4.5)	0 (0.0)	a, b > 0.05
A39T	7 (21.2)	4 (12.1)	6 (13.6)	4 (9.1)	
G45D	8 (24.2)	1 (3.0)	5 (11.3)	2 (4.5)	
H105Y	16 (48.5)	1 (3.0)	26 (59.1)	11 (25.0)	
A39T/ F62L	0 (0.0)	0 (0.0)	1 (2.3)	0 (0.0)	
A40D/T86A	0 (0.0)	0 (0.0)	1 (2.3)	0 (0.0)	
A40D/T86A/H105Y	0 (0.0)	0 (0.0)	1 (2.3)	1 (2.3)	
D79N/T86A/H105Y	0 (0.0)	0 (0.0)	1 (2.3)	1 (2.3)	
T86A/ H105Y	0 (0.0)	0 (0.0)	1 (2.3)	0 (0.0)	
Mutation patterns in PBP2					
II or XIV	3 (9.1)	0 (0.0)	4 (9.1)	0 (0.0)	a, b > 0.05
V	6 (18.2)	1 (3.0)	8 (18.2)	3 (6.8)	
VII	0 (0.0)	0 (0.0)	1 (2.3)	0 (0.0)	
VIII	0 (0.0)	0 (0.0)	1 (2.3)	1 (2.3)	
XII	2 (6.1)	0 (0.0)	2 (4.6)	1 (2.3)	
XIII	2 (6.1)	0 (0.0)	0 (0.0)	0 (0.0)	
XVII	2 (6.1)	1 (3.0)	0 (0.0)	0 (0.0)	
XVIII	5 (15.2)	2 (6.1)	13 (29.4)	10 (22.6)	
XXI	7 (21.2)	1 (3.0)	8 (18.2)	3 (6.8)	
XXVII	4 (12.2)	0 (0.0)	6 (13.6)	1 (2.3)	
New 1^d^	1 (3.0)	0 (0.0)	0 (0.0)	0 (0.0)	
New 2^d^	1 (3.0)	1 (3.0)	0 (0.0)	0 (0.0)	
New 3^d^	0 (0.0)	0 (0.0)	1 (2.3)	0 (0.0)	
23rRNA/*mtrR*/penA mutation patterns					
WT/A-;H105Y/XVIII	2 (6.1)	0 (0.0)	4 (9.1)	3 (6.8)	a, b > 0.05
WT/A-;H105Y/V	6 (18.2)	1 (3.0)	6 (13.6)	3 (6.8)	
WT/A-;H105Y/XXI	2 (6.1)	0 (0.0)	6 (13.6)	2 (4.5)	
WT/A-;G45D/XXI	4 (12.1)	3 (9.1)	0 (0.0)	0 (0.0)	
WT/A-;A39T/XVIII	3 (9.1)	3 (9.1)	5 (11.4)	3 (6.8)	
C2611T^All^/A-;H105Y/XVIII	0 (0.0)	0 (0.0)	2 (4.5)	2 (4.5)	
C2611T^All^/A-;G45D/XXI	0 (0.0)	0 (0.0)	2 (4.5)	2 (4.5)	
Other patterns	16 (48.5)	2 (6.1)	17 (38.6)	2 (4.5)	

*AZM* azithromycin, *CRO* ^*D*^ decreased susceptibility to ceftriaxone, *CRO* ^*S*^ susceptible to ceftriaxone, *C2611T* ^*S*^ mutation C2611T in a single allele, *C2611T* ^*All*^ C2611T mutations in all four alleles, ^a^Adenine (A) deletion in the 13-bp inverted repeat (5’-AAAAAGACTTTTT-3’) within the -35 to -10 positions of the *mtrR* promoter. ^b^Using *mtrR* from *N. gonorrhoeae* FA1090 as a template. ^c^Including CRO^S^ and CRO^D^; ^d^New polymorphism patterns of PBP2: New 1 (A501V, F504L, A510V, A516G, H541N, P552V, I556V, I566V), New 2 (F504L, A510V, A516G, Y554H), New 3 (A501V, F504L, A510V, A516G, H541N, I566V). a: the AZM-LLR isolates were compared to the AZM-MLR isolates; b: the combined AZM-LLR/CRO^D^ isolates were compared to the combined AZM-MLR/CRO^D^ isolates; *ND* not determined (because of a small number of isolates)

### 23S rRNA, *mtrR* and penA mutations in AZM-R *N. gonorrhoeae* isolates

Thirty-two of the 33 AZM-LLR isolates (97.0 %) contained the wild-type sequence in domain V of all four 23S rRNA alleles, except one isolate (3.0 %, 1/33) that contained mutation C2611T (Escherichia coli numbering; also known as C2599T, *N. gonorrhoeae* numbering) in a single allele (Table 2 and Additional file 1: Table S1, S2). Thirty-eight of the 44 AZM-MLR isolates (86.4 %) contained the wild-type sequence in all four 23S rRNA alleles, whereas only one isolate (2.2 %, 1/44; its AZM MIC **=** 2 mg/L) contained mutation C2611T in a single allele and five isolates (11.4 %, 5/44; their AZM MICs **=** 4, 8, 8, 8 and 64 mg/L, respectively) contained mutation C2611T in all four alleles. All seven AZM-R isolates that contained the mutation C2611T showed reduced susceptibility to ceftriaxone (MIC ≥ 0.125 mg/L).

The presence of a single nucleotide deletion (A) in the 13 bp inverted repeat of the *mtrR* promoter did not different between AZM-LLR (87.9 %, 29/33) and AZM-MLR (95.5 %, 42/44) isolates (*P* > 0.05) (Table 2 and Additional file 1: Table S1, S2). A wild type (WT) in the coding region of *mtrR* only displayed in combined AZM-LLR/susceptibility to ceftriaxone (CRO^S^) (*n* = 2) and combined AZM-MLR/CRO^S^ (*n* = 2), but not in combined AZM-LLR/CRO^D^ or combined AZM-MLR/CRO^D^ isolates. The combined mutations (double or triple) such as A39T/F62L, A40D/T86A, T86A/H105Y, A40D/T86A/H105Y or D79N/T86A/H105Y were only present in AZM-MLR isolates (Table 2).

All the AZM-R isolates (*n* = 77) in this study had an Asp-346 insertion and contained F504L, A510V and A516G mutations in PBP2 (data shown in Additional file 1: Table S1-S3). Thirteen mutation patterns in this study included three new mutation patterns (New 1: A501V, F504L A501V, A516G, H541N, P552V, I556V, I566V; New 2: F504L, A501V, A516G, Y554H; New 3: A501V, F504L, A510V, A516G, H541N, I566V) were shown in Table 2. The patterns V (18.2 %), XVIII (15.2 %) and XXI (21.2 %) were the most prevalent in AZM-LLR isolates, similar to the most prevalent patterns (V, 18.2 %; XVIII, 29.4 %; XXI, 18.2 %) in AZM-MLR isolates. No different mutation patterns were found between AZM-LLR isolates and AZM-MLR isolates or between combined AZM-LLR/CRO^D^ isolates and combined AZM-MLR/CRO^D^ isolates (both *P* > 0.05).

In addition, combined 23S rRNA/*mtrR*/penA mutation patterns had no significant association with AZM-MLR isolates or combined AZM-MLR/CRO^D^ isolates (Table 2).

### NG-MAST and phylogenetic analysis based on *porB* and *tbpB* DNA sequences

A total of 67 STs (29 STs in AZM-LLR isolates and 38 STs in AZM-MLR isolates) were identified by NG-MAST among the 77 AZM-R isolates, of which 30 STs (15 STs in AZM-LLR isolates and 15 STs in AZM-MLR isolates) were first identified by the current study (Table 3 and Additional file 1: Table S1, S2 and S3). The most prevalent STs were ST1766 (*n* = 3), ST1866 (*n* = 2), and ST421 (*n* = 2) in AZM-LLR isolates and ST1766 (*n* = 3), ST6987 (*n* = 2), ST1055 (*n* = 2), ST304 (*n* = 2), and a new ST consisting of *porB* allele 822 and *tbpB* allele 156 (*n* = 2) in AZM-MLR isolates. Of the remaining STs, most were represented by a single isolate. The phylogenetic tree, built from DNA sequences of *por* (490 bp) and *tbpB* (390 bp), displayed the significant diversity of strains with 29 different STs in AZM-LLR isolates (Additional file 2: Figure S1), as well as 38 different STs in AZM-MLR isolates (Additional file 3: Figure S2).

**Table 3:** NG-MAST STs and *N. gonorrhoeae* isolated with AZM-R and CRO^D^ identified in Guangzhou in 2009-2013

	AZM-LLR (*n* = 33)		AZM-MLR (*n* = 44)	
	Total^b^	CRO^D^	Total^b^	CRO^D^
NG-MAST ST	*n* (%)	*n* (%)	*n* (%)	*n* (%)
270	0 (0.0)	0 (0.0)	1 (2.3)	1 (2.3)
304	0 (0.0)	0 (0.0)	2 (4.5)	0 (0.0)
421	2 (6.1)	0 (0.0)	0 (0.0)	0 (0.0)
1053	1 (3.0)	0 (0.0)	1 (2.3)	1 (2.3)
1055	0 (0.0)	0 (0.0)	2 (4.5)	0 (0.0)
1056	0 (0.0)	0 (0.0)	1 (2.3)	0 (0.0)
1412	1 (3.0)	0 (0.0)	0 (0.0)	0 (0.0)
1731	1 (3.0)	0 (0.0)	0 (0.0)	0 (0.0)
1766	3 (9.1)	0 (0.0)	3 (6.8)	0 (0.0)
1866	2 (6.1)	0 (0.0)	1 (2.3)	0 (0.0)
1972	1 (3.0)	1 (3.0)	0 (0.0)	0 (0.0)
2103	1 (3.0)	0 (0.0)	0 (0.0)	0 (0.0)
2384	0 (0.0)	0 (0.0)	1 (2.3)	1 (2.3)
3079	1 (3.0)	0 (0.0)	0 (0.0)	0 (0.0)
3252	0 (0.0)	0 (0.0)	1 (2.3)	1 (2.3)
3356	0 (0.0)	0 (0.0)	1 (2.3)	1 (2.3)
3460	1 (3.0)	0 (0.0)	0 (0.0)	0 (0.0)
4313	0 (0.0)	0 (0.0)	1 (2.3)	1 (2.3)
4539	1 (3.0)	0 (0.0)	0 (0.0)	0 (0.0)
5061	0 (0.0)	0 (0.0)	1 (2.3)	1 (2.3)
5062	0 (0.0)	0 (0.0)	1 (2.3)	0 (0.0)
5179	1 (3.0)	1 (3.0)	0 (0.0)	0 (0.0)
5990	0 (0.0)	0 (0.0)	1 (2.3)	0 (0.0)
6987	0 (0.0)	0 (0.0)	2 (4.5)	1 (2.3)
7101	0 (0.0)	0 (0.0)	1 (2.3)	1 (2.3)
8776	1 (3.0)	0 (0.0)	0 (0.0)	0 (0.0)
9176	0 (0.0)	0 (0.0)	1 (2.3)	1 (2.3)
9944	1 (3.0)	0 (0.0)	0 (0.0)	0 (0.0)
10199	0 (0.0)	0 (0.0)	1 (2.3)	1 (2.3)
10205	0 (0.0)	0 (0.0)	1 (2.3)	1 (2.3)
10337	0 (0.0)	0 (0.0)	1 (2.3)	0 (0.0)
10352	0 (0.0)	0 (0.0)	1 (2.3)	0 (0.0)
10359	0 (0.0)	0 (0.0)	1 (2.3)	0 (0.0)
11181	0 (0.0)	0 (0.0)	1 (2.3)	0 (0.0)
New^a^	15 (45.5)	4 (12.1)	16 (36.4)	7 (15.9)

*AZM* azithromycin, *CRO* ^*D*^decreased susceptibility to ceftriaxone, *CRO* ^*S*^ susceptible to ceftriaxone, ^a^Not found in the NG-MAST database (http://www.ng-mast.net); ^b^Including CRO^S^ and CRO^D^

## Discussion

The present study combines antimicrobial susceptibility determinations with molecular-based analysis of AZM-R in *N. gonorrhoeae* isolated from 2009 to 2013 in Guangzhou, China. As observed in other countries worldwide [9, 13, 18], an upward shift in MIC by year and a high percentage of AZM-R were identified in this study, which was also in line with a previous report in two Chinese cities, Nanjing and Chongqing, between 2008 and 2009 [5]. The WHO recommends discontinuation of the empirical use of an antibiotic once 5 % of locally acquired gonococcal isolates are resistant [19]. Accordingly, AZM has not been recommended as a monotherapy for gonococcal urethritis or cervicitis in China, as well as in many other countries worldwide. To improve treatment efficacy and to delay the further selection of cephalosporin-resistant *N. gonorrhoeae*, most current guidelines now recommend ESCs and AZM in combination as the first-line treatment for gonorrhea [2–4, 9]. However, more recently, AZM-R *N. gonorrhoeae* has been rarely and sporadically reported to also have reduced susceptibility to ESCs [9, 13], threatening the future efficacy of current therapeutic recommendations. Similarly, the present study showed that the prevalence of combined AZM-MLR/CRO^D^ isolates significantly increased as compared with that in all identified *N. gonorrhoeae* isolates.

Previous studies investigating AZM-R and CRO^D^ have focused on the mutations of three genes: 23S rRNA, *mtrR* and penA [9–12]. Mutations at various positions within the central loop of domain V of the 23S rRNA gene are thought to lead to resistance by reducing the ability of the 23S rRNA protein to bind the antibiotics such as AZM [10]. Previous reports have shown that the mutation C2611T in any of the four alleles of the 23S rRNA can result in a lower level of resistance to AZM [10]. In addition, the mutation A2059G (Escherichia coli numbering) in at least three of the four alleles can confer AZM-HLR, and AZM-sensitive isolates containing this single allele mutation can quickly develop high-level resistance in the presence of erythromycin [10]. In the current study, only seven AZM-R isolates contained mutation C2611T, whereas the remaining large number of AZM-R isolates contained four wild-type alleles. These findings contrast, however, with other reports from Canada [9], the United States [11, 20] and the United Kingdom [10], which most of the AZM-MLR isolates contained this mutation was more than 80 %. Notably, all the above-mentioned seven AZM-R isolates that contained the mutation C2611T showed reduced susceptibility to ceftriaxone. The mutation A2059G, which is linked to AZM-HLR, was not detected in the present study. In the *mtrR* gene, specific mutations in the promoter or coding region can lead to decrease the MtrCDE efflux pump repression and subsequently increase export of the antimicrobials [20]. Loss of the MtrCDE efflux pump can significantly increase susceptibility to AZM, ESCs, penicillin, ciprofloxacin, and solithromyc in *N. gonorrhoeae* isolates [21]. In this study, a single nucleotide deletion in the mtrR promoter showed over 85 % in AZMR-R isolates, but not significantly difference between combined AZMR-R/CRO^S^ isolates and combined AZMR-R/CRO^D^ isolates, in line with a previous report that such a deletion may not implicate in CRO^D^ [12]. H105Y, the most prevalent single mutation, was not significantly difference in AZM-LLR isolates as compared to AZM-MLR isolates. Interestingly, the combined mutation in *mtrR* was only observed in AZM-MLR isolates, but not in in AZM-LLR isolates. Mutation in penA has been associated with decreased susceptibility or resistance to a number of beta-lactam agents, especially to ESCs [22]. The mutations G545S, I312M, and V316T in mosaic alleles of PBP2 were early suggested as important for the increased ESCs resistance [23]. However, a recent study revealed that these three mutations had only a little to effect on resistance, and their capacity to increase resistance to ESCs was dependent on the presence of other mutations in the mosaic alleles [24]. Mutation patterns XIII, XVII and XVIII have been reported to only present in CRO^D^ isolates in China [12] and Australia [22]. These three patterns contained G542S, P551S and/or P551L mutations, which have been associated with increased MIC of CRO [25]. However, their effects on the ceftriaxone MIC have not yet been proven by site-directed penA mutations in isogenic strain backgrounds [26] and the present study showed that these three patterns were present in both CRO^S^ and CRO^D^ isolates (data not shown). The most prevalent mutation pattern in AZMR-R isolates from Canada was XXXIV, which was showed to AZM-R together with reduced susceptibility to ESCs [9]. By comparison, this study revealed the most prevalent mutation patterns (V, XVIII and XXI) were the same in AZMR-LLR, AZMR-MLR, and combined AZMR-R/CRO^D^ isolates, respectively. Overall, the results of our study showed that the mutations of these three genes had the considerable diversity, and that either one of these three genes alone or combined mutation patterns of these three genes cannot account for AZM-MLR or combined AZM-R/CRO^D^ in our *N. gonorrhoeae* isolates.

NG-MAST analysis and the resulting development of a phylogenetic tree in this study also indicated a high degree of genetic diversity of *N. gonorrhoeae* in Guangzhou from 2009 to 2013. This observation may be due to the highly limited epidemiologic information of this study because our data do not represent the whole country, or the flow of large numbers of temporary population in Guangzhou, which lead to great opportunity for transport of new STs [27]. Also, AZM is extensively used in China [5] and has long elimination half-life. Thus we postulate that the diversity of STs developed, in part, in response to antibiotic pressure. Some STs that were observed in the current study (ST3356, ST1866, ST1766, and ST4313) had been noted in a previous report of emerging AZM-R *N. gonorrhoeae* in Nanjing and Chongqing, China [5], but these did not include STs (ST470, ST649, ST3158, ST1704, ST359, and ST696) that were reported previously in other countries and have been shown to account for most of the resistance or high-level resistance to AZM [9, 18, 28–30].

## Conclusions

AZM-R *N. gonorrhoeae* isolates are increasing and, in combination with CRO^D^, have now been detected in Guangzhou. The combined AZM-R/CRO^D^ in *N. gonorrhoeae* is a relatively new phenomenon, and the mechanism for the emergence of these AZM-R/CRO^D^ isolates remains unknown and needs further studied. Therefore, antimicrobial susceptibility/resistance and the molecular epidemiology of AZM-R and/or CRO^D^ deserve continuous surveillance, which will be critical in providing valuable information for current and future therapeutic options in China.

### Availability of data and materials

The sequencing datasets of porB, tbpB, 23S rRNA, mtrR and penA genes supporting the conclusions of this article are available in the Dryad Digital Repository: http://dx.doi.org/10.5061/dryad.cp3d8.

## Additional files

Additional file 1:
**Table S1.** Characteristics of AZM-LLR *N. gonorrhoeae* isolates identified in Guangzhou in 2009-2013 (*n* = 33). **Table S2.** Characteristics of AZM-MLR *N. gonorrhoeae* isolates identified in Guangzhou in 2009-2013 (n = 44). **Table S3.** The classification of mutation patterns of the PBP2 proteins based on amino acids 500 to 570. (DOCX 46.2 kb) Additional file 2:
**Figure S1.** A neighbor-joining phylogenetic tree was constructed using the concatenated sequences of *por* (490 bp) and *tbpB* (390 bp) alleles, which were identified by the NG-MAST method, from 33 AZM-LLR *N. gonorrhoeae* isolates in Guangzhou, China in 2009–2013. (DOCX 1475 kb) Additional file 3:
**Figure S2.** A neighbor-joining phylogenetic tree was constructed using the concatenated sequences of *por* (490 bp) and *tbpB* (390 bp) alleles, which were identified by the NG-MAST method, from 44 AZM-MLR *N. gonorrhoeae* isolates in Guangzhou, China in 2009–2013. (DOCX 1372 kb)

## Abbreviations

AZM: azithromycin
AZM-HLR: azithromycin high-level resistant
AZM-LLR: azithromycin low-level resistant
AZM-MLR: azithromycin middle-level resistant
AZM-R: azithromycin-resistant
CRO^D^: decreased susceptibility to ceftriaxone
CRO^S^: susceptibility to ceftriaxone
ESCs: extended-spectrum cephalosporins
MIC: minimum inhibitory concentration
NG-MAST: *N. gonorrhoeae* multi-antigen sequence typing
PBP2: penicillin-binding protein 2
STI: sexually transmitted infection.
