# Supplementary material for: Azithromycin-resistant Neisseria gonorrhoeae isolates in Guangzhou, China (2009–2013): coevolution with decreased susceptibilities to ceftriaxone and genetic characteristics
Source: BMC Infect Dis. 2016 Apr 14;16:152. doi: 10.1186/s12879-016-1469-3 (PMC4832481; doi:10.1186/s12879-016-1469-3)
Supplement: Additional file 1: — Table S1. Characteristics of AZM-LLR N. gonorrhoeae isolates identified in Guangzhou in 2009-2013 (n = 33). Table S2. Characteristics of AZM-MLR N. gonorrhoeae isolates identified in Guangzhou in 2009-2013 (n = 44). Table S3. The classification of mutation patterns of the PBP2 proteins based on amino acids 500 to 570. (DOCX 46.2 kb) [file 12879_2016_1469_MOESM1_ESM.docx]

**Table S1 Characteristics of AZM-LLR *N. gonorrhoeae* isolates identified in Guangzhou in 2009-2013 (n = 33).**

| Strain  Code | MIC (mg/L) 23S mtrR PBP2  AZM CRO rRNA deletion^#^ alterations patterns | | | | | | NG-MAST  ST por tbpB | | |
| --- | --- | --- | --- | --- | --- | --- | --- | --- | --- |
| GZ-09-1 | 1 | D(0.125) | All WT | Yes | A39T | XVII | 5179 | 3137 | 156 |
| GZ-09-4 | 1 | S(0.031) | All WT | Yes | G45D | XXI | 1731 | 4 | 186 |
| GZ-09-8 | 1 | S(0.031) | All WT | No | WT* | New 1 | No** | 2217 | 33 |
| GZ-09-13 | 1 | D(0.125) | All WT | Yes | A39T | XVIII | No** | 489 | 119 |
| GZ-09-25 | 1 | S(0.008) | All WT | Yes | WT* | Ⅱ or XIV | 1866 | 581 | 33 |
| GZ-09-27 | 1 | S(0.031) | All WT | Yes | H105Y | XII | 8776 | 4199 | 479 |
| GZ-09-56 | 1 | D(0.125) | All WT | Yes | A39T | New 2 | 1972 | 130 | 156 |
| GZ-09-67 | 1 | S(0.031) | All WT | Yes | H105Y | XII | No** | 4258 | No** |
| GZ-09-70 | 1 | S(0.008) | All WT | Yes | G45D | XXI | 421 | 206 | 156 |
| GZ-09-71 | 1 | S(0.031) | All WT | Yes | H105Y | XXVII | 1766 | 1132 | 33 |
| GZ-09-74 | 1 | D(0.125) | All WT | Yes | A39T | XVIII | No** | 182 | 328 |
| GZ-09-77 | 1 | S(0.031) | All WT | Yes | H105Y | XXI | 1766 | 1132 | 33 |
| GZ-09-81 | 1 | S(0.031) | All WT | Yes | H105Y | XXVII | 1766 | 1132 | 33 |
| GZ-09-82 | 1 | S(0.063) | All WT | Yes | G45D | XXI | 421 | 206 | 156 |
| GZ-09-85 | 1 | S(0.008) | All WT | No | A39T | Ⅱ or XIV | No** | 90 | No** |
| GZ-09-87 | 1 | S(0.031) | All WT | Yes | H105Y | XXI | 3079 | 90 | 484 |
| GZ-09-88 | 1 | S(0.031) | All WT | No | A39T | XVIII | 3460 | 2053 | 107 |
| GZ-10-15 | 1 | S(0.031) | All WT | Yes | H105Y | V | 1053 | 182 | 33 |
| GZ-10-16 | 1 | D(0.25) | All WT | Yes | H105Y | V | No** | No** | 738 |
| GZ-10-17 | 1 | S(0.031) | All WT | Yes | H105Y | XVIII | 9944 | 1123 | 4 |
| GZ-10-36 | 1 | S(0.031) | All WT | No | G45D | XIII | 4539 | 4 | 752 |
| GZ-10-43 | 1 | S(0.016) | All WT | Yes | H105Y | V | No** | 1466 | No** |
| GZ-10-44 | 1 | D(0.125) | C2611T^S^ | Yes | G45D | XXI | No** | 1275 | 156 |
| GZ-10-45 | 1 | S(0.031) | All WT | Yes | G45D | XXVII | 1866 | 581 | 33 |
| GZ-11-81 | 1 | S(0.063) | All WT | Yes | A39T | XVII | 2103 | 1323 | 4 |
| GZ-12-23 | 1 | S(0.031) | All WT | Yes | G45D | XXI | 1412 | 4 | 10 |
| GZ-12-88 | 1 | S(0.063) | All WT | Yes | H105Y | XIII | No** | 2261 | 33 |
| GZ-13-23 | 1 | S(0.015) | All WT | Yes | G45D | XXVII | No** | No** | 156 |
| GZ-13-26 | 1 | S(0.031) | All WT | Yes | H105Y | Ⅱ or XIV | No** | No** | 110 |
| GZ-13-27 | 1 | S(0.031) | All WT | Yes | H105Y | V | No** | 5541 | 33 |
| GZ-13-39 | 1 | S(0.031) | All WT | Yes | H105Y | V | No** | No** | 33 |
| GZ-13-45 | 1 | S(0.063) | All WT | Yes | H105Y | V | No** | 1445 | 738 |
| GZ-13-51 | 1 | S(0.063) | All WT | Yes | H105Y | XVIII | No** | No** | 4 |

AZM, azithromycin; CRO, ceftriaxone; R, resistant; D, decreased susceptible; S, susceptible; C2611T^All^, C2611T mutations in all four alleles; C2611T^S^, a C2611T mutation in a single allele; ^#^ Adenine (A) deletion in the 13-bp inverted repeat (5’-AAAAAGACTTTTT-3’) within the -35 to -10 positions of the *mtrR* promoter; *Using *mtrR* from *N. gonorrhoeae* FA1090 as a template; ** Not found in the NG-MAST database (<http://www.ng-mast.net>).

**Table S2 Characteristics of AZM-MLR *N. gonorrhoeae* isolates identified in Guangzhou in 2009-2013 (n = 44).**

| Strain  Code | MIC (mg/L) 23S mtrR PBP2  AZM CRO rRNA deletion^#^ alterations patterns | | | | | | NG-MAST  ST por tbpB | | |
| --- | --- | --- | --- | --- | --- | --- | --- | --- | --- |
| GZ-09-48 | 2 | S(0.063) | All WT | Yes | A39T | XVIII | No** | No** | 29 |
| GZ-09-52 | 2 | D(0.125) | All WT | Yes | H105Y | XVIII | No** | 1264 | 186 |
| GZ-09-80 | 8 | S(0.015) | All WT | Yes | H105Y | XXI | 1766 | 1132 | 33 |
| GZ-09-86 | 8 | D(0.25) | C2611T^All^ | Yes | H105Y | XXVII | 4313 | 130 | 526 |
| GZ-10-20 | 4 | D(0.125) | C2611T^All^ | Yes | H105Y | XVIII | 3356 | 2035 | 526 |
| GZ-10-32 | 2 | D(0.125) | C2611T^S^ | Yes | A39T | XVIII | No** | 4187 | 470 |
| GZ-10-77 | 8 | D(0.125) | C2611T^All^ | Yes | G45D | XXI | No** | 822 | 156 |
| GZ-10-78 | 8 | D(0.125) | C2611T^All^ | Yes | G45D | XXI | No** | 822 | 156 |
| GZ-11-24 | 8 | S(0.016) | All WT | Yes | H105Y | XXI | No** | 164 | No** |
| GZ-11-39 | 8 | S(0.063) | All WT | Yes | H105Y | XII | 11181 | 2978 | 1336 |
| GZ-11-43 | 2 | D(0.125) | All WT | Yes | H105Y | V | 7101 | 609 | 367 |
| GZ-11-50 | 8 | S(0.032) | All WT | Yes | G45D | Ⅱor XIV | 1866 | 581 | 33 |
| GZ-11-51 | 2 | D(0.125) | All WT | Yes | A39T | XVIII | No** | No** | 137 |
| GZ-11-56 | 8 | S(0.032) | All WT | Yes | H105Y | V | No** | No** | 738 |
| GZ-11-57 | 2 | S(0.032) | All WT | Yes | H105Y | New 3 | 1056 | 609 | 186 |
| GZ-11-84 | 2 | S(0.016) | All WT | Yes | H105Y | XXI | No** | 90 | 737 |
| GZ-11-85 | 2 | D(0.5) | All WT | Yes | H105Y | XII | 2384 | 1208 | 33 |
| GZ-12-3 | 2 | D(0.125) | All WT | Yes | A40D,T86A,H105Y | XXI | 10199 | 5979 | 10 |
| GZ-12-4 | 2 | S(0.063) | All WT | No | A39T,F62L | XXI | 5990 | 3580 | 156 |
| GZ-12-6 | 8 | S(0.063) | All WT | Yes | H105Y | XXVII | 1766 | 1132 | 33 |
| GZ-12-8 | 4 | S(0.063) | All WT | Yes | A39T | XVIII | 6987 | 4195 | 1308 |
| GZ-12-36 | 4 | S(0.063) | All WT | Yes | H105Y | XVIII | 10337 | 147 | 709 |
| GZ-12-39 | 4 | S(0.063) | All WT | Yes | H105Y | XXI | 10352 | 866 | 186 |
| GZ-12-45 | 8 | D(0.125) | All WT | Yes | H105Y | V | 10205 | 5985 | 1495 |
| GZ-12-46 | 2 | D(0.125) | All WT | Yes | D79N,T86A,H105Y | XVIII | 9176 | 4197 | 110 |
| GZ-12-49 | 4 | D(0.125) | All WT | Yes | H105Y | XVIII | 6987 | 4195 | 1308 |
| GZ-12-56 | 8 | D(0.125) | All WT | Yes | H105Y | V | 1053 | 182 | 33 |
| GZ-12-59 | 4 | D(0.125) | All WT | Yes | H105Y | VIII | 5061 | 2978 | 1058 |
| GZ-12-68 | 2 | S(0.032) | All WT | Yes | WT* | VII | No** | 2978 | No** |
| GZ-12-70 | 8 | S(0.032) | All WT | Yes | H105Y | V | 10359 | 1447 | 10 |
| GZ-12-85 | 4 | S(0.032) | All WT | Yes | H105Y | V | No** | 876 | 1036 |
| GZ-12-93 | 8 | D(0.125) | All WT | Yes | A39T | XVIII | No** | 5997 | 479 |
| GZ-12-99 | 8 | S(0.063) | All WT | No | A40D,T86A | XXVII | No** | 3215 | 913 |
| GZ-12-100 | 8 | D(0.125) | All WT | Yes | A39T | XVIII | No** | 952 | 10 |
| GZ-13-10 | 32 | S(0.032) | All WT | Yes | H105Y | Ⅱor XIV | No** | No** | 4 |
| GZ-13-30 | 2 | S(0.063) | All WT | Yes | H105Y | Ⅱor XIV | 5062 | 3029 | 1036 |
| GZ-13-40 | 32 | S(0.032) | All WT | Yes | H105Y | Ⅱor XIV | No** | No** | 479 |
| GZ-13-44 | 64 | D(0.125) | C2611T^All^ | Yes | H105Y | XVIII | 270 | 206 | 33 |
| GZ-13-47 | 2 | D(0.125) | All WT | Yes | H105Y | XVIII | 3252 | 4 | 156 |
| GZ-13-70 | 16 | S(0.063) | All WT | Yes | WT* | V | 1055 | 581 | 4 |
| GZ-13-74 | 2 | S(0.015) | All WT | Yes | T86A, H105Y | XXVII | 1766 | 1132 | 33 |
| GZ-13-76 | 16 | S(0.032) | All WT | Yes | WT | V | 1055 | 581 | 4 |
| GZ-13-82 | 16 | S(0.015) | All WT | Yes | G45D | XXVII | 304 | 4 | 75 |
| GZ-13-94 | 16 | S(0.015) | All WT | Yes | G45D | XXVII | 304 | 4 | 75 |

AZM, azithromycin; CRO, ceftriaxone; R, resistant; D, decreased susceptible; S, susceptible; C2611T^All^, C2611T mutations in all four alleles; C2611T^S^, mutation C2611T in a single allele; ^#^ Adenine (A) deletion in the 13-bp inverted repeat (5’-AAAAAGACTTTTT-3’) within the -35 to -10 positions of the *mtrR* promoter; *Using *mtrR* from *N. gonorrhoeae* FA1090 as a template; ** Not found in the NG-MAST database (<http://www.ng-mast.net>).

**Table S3 The classification of mutation patterns of the PBP2 proteins based on amino acids 500 to 570.**

| Mutation patterns | Amino acid mutations  from 500 to 570 |
| --- | --- |
| Ӏ |  |
| Ⅱ or XIV | F504L, A510V, A516G |
| Ⅲ | F504L, A510V, A516G, I566V |
| Ⅳ | F504L, A510V, A516G, G542S |
| Ⅴ | F504L, A510V, A516G, G542S, I566V |
| Ⅵ | F504L, A510V, P552V |
| Ⅶ | A501V, F504L, A510V, A516G, G542S |
| Ⅷ | A501V, F504L, A510V, G542S, P551L |
| Ⅸ | F504L, A510V, A516G, P551L |
| Ⅹ | F504L, A510V, N512Y, H541N, G545, A549T, P552V, K555Q, I556V, I566V |
| Ⅺ | A501V, F504L, A510V, A516G, P551L |
| Ⅻ | F504L, A510V, A516G, P551S |
| XIII | A501V, F504L, A510V, A516G, P551S |
| XIV | F504L, A510V, A516G, H541N |
| XV | H541N |
| XVII | A501V, F504L, A510V, A516G, G542S, I566V |
| XVIII | A501T, F504L, A510V, A516G,G542S, I566V |
| XIX or XX | F504L, A510V, A516G, H541N, I566V |
| XXI | A501V, F504L, A510V, A516G, H541N,P552V, K555Q, I556V, I566V |
| XXII | F504L, A510V, A516G, H541N, P552V, K555Q , I556V, I566V |
| XXIII | F504L, A510V, N512Y, H541N, A549T, P552V, K555L, I556V, I566V |
|  |  |
| XXV | A501T, F504L, A510V, A516G, G542S |
| XXVI | A501V, F504L, A510V, A516G, G542S, P551S, I566V |
| XXVII | A501V, F504L, A510V, A516G |
| XXVIII | A501V, F504L, A510V, A516G, I566V |
| XXIX | A501V, F504L, A510V, A516G, H541N |
| XXX | A501T, F504L, A510V, A516G, G542S, I563V |

The sequences are classified into different mutation patterns (patterns I to XXIII and XXV to XXXIII) based on amino acids 500 to 570 and are aligned with a GenBank *N. gonorrhoeae* sequence (accession number M32091). Sequence patterns I to X were previously described by Ito et al. [Antimicrob Agents Chemother. 2005 Jan;49(1):137-43], XI to XXIII by Whiley et al. [Antimicrob Agents Chemother. 2007 Sep;51(9):3111-6], and XXV to XXX by Liao et al. [J Antimicrob Chemother 2011; 66: 1016–1023].
