# Supplementary material for: Azithromycin-resistant Neisseria gonorrhoeae isolates in Guangzhou, China (2009–2013): coevolution with decreased susceptibilities to ceftriaxone and genetic characteristics
Source: BMC Infect Dis. 2016 Apr 14;16:152. doi: 10.1186/s12879-016-1469-3 (PMC4832481; doi:10.1186/s12879-016-1469-3)
Supplement: Additional file 3: — Figure S2. A neighbor-joining phylogenetic tree was constructed using the concatenated sequences of por (490 bp) and tbpB (390 bp) alleles, which were identified by the NG-MAST method, from 44 AZM-MLR N. gonorrhoeae isolates in Guangzhou, China in 2009–2013. (DOCX 1372 kb) [file 12879_2016_1469_MOESM3_ESM.docx]

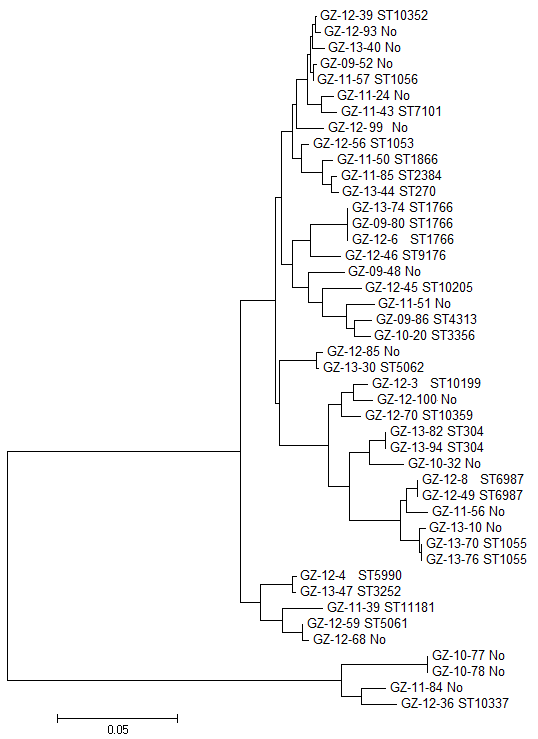


**Figure S2 A neighbor-joining phylogenetic tree was constructed using the concatenated sequences of *por* (490 bp) and *tbpB* (390 bp) alleles, which were identified by the NG-MAST method, from 44 AZM-MLR *N. gonorrhoeae* isolates in Guangzhou, China in 2009–2013.**
